# O Jejum Intermitente e a Redução da Pressão Arterial: Mecanismos Relacionados

**DOI:** 10.36660/abc.20230265

**Published:** 2023-06-02

**Authors:** Rui Póvoa

**Affiliations:** 1 Universidade Federal de São Paulo São Paulo SP Brasil Universidade Federal de São Paulo, São Paulo, SP – Brasil

**Keywords:** Doenças Cardiovasculares/mortalidade, Obesidade, Hipertensão, Jejum, Restrição Calórica, Perda Peso

A obesidade, junto com uma dieta inadequada são fatores de risco modificáveis com importante peso no aumento das doenças cardiovasculares. Apresentam um risco atribuível estimado de 13% na mortalidade cardiovascular.^1^ Existem várias intervenções dietéticas que podem melhorar este risco, principalmente a restrição calórica, que está ligada a redução do peso, melhoras da sensibilidade a insulina e redução da pressão arterial.^2 , 3^

O jejum intermitente (JI) é uma intervenção dietética semelhante a restrição calórica, pois utiliza o princípio da restrição da ingesta de alimentos. Os dois tipos mais abrangentes são o jejum diurno alternativo (24 horas de jejum seguidos de um período de 24 horas de alimentação) e o jejum com restrição de tempo (variações de 16-20 horas).^4^

Alguns estudos em humanos mostraram benefícios cardiovasculares e redução pressórica com a prática do JI. Sutton et al., em 2018 avaliando a redução da pressão arterial com o JI de 18 horas encontraram após seis semanas redução de 11± 4 mmHg para a pressão arterial sistólica e 10 ± 4 mmHg para a diastólica.^5^

Os mecanismos que propõem explicar estas variações pressóricas são complexos e multifatoriais, como a própria gênese da hipertensão. Entretanto a diminuição da atividade simpática e o aumento do tônus parassimpático, além da redução da atividade do sistema renina angiotensina aldosterona (SRAA) são os fenômenos mais concorrentes para esta queda pressórica.^6^ Os estudos experimentais em ratos, onde é possível controlar melhor as variáveis, apontam neste sentido, onde o JI corrige o desequilíbrio autonômico, reduzindo a pressão, além de elevação do tônus vagal associado a menores níveis de citocinas inflamatórias.^7^

Entretanto estudos controlados avaliando estes sistemas e suas interações em humanos são bastante escassos, com resultados que não permitem uma explicação coerente.

Dermirci et al.,^8^ analisaram a influência do JI na pressão arterial com extensa avaliação dos marcadores de atividade do SRAA e da atividade simpática.^8^ O trabalho envolveu uma quantidade significante de pacientes hipertensos com avaliações antes e depois do JI e os resultados foram muito interessantes, principalmente nos marcadores relacionados com o desenvolvimento da hipertensão. Após 15-16 horas de jejum durante 30 dias encontraram reduções significantes da enzima conversora da angiotensina I (ECA) e da angiotensina II (Ang-II) e aumento, também significante, da angiotensina I (ang-I). Aliada a estes resultados houve redução pressórica com significância estatística, tanto da pressão arterial sistólica quando da diastólica, inclusive a noite. Resultados coerentes que vão ao encontro dos aspectos fisiopatológicos da hipertensão arterial.

Sabemos que a perda de peso corpóreo é um fator importante para a redução pressórica sendo que o JI, a curto prazo, pode proporcionar estes benefícios.

Evidentemente os efeitos do JI no estudo de Dermirci et al.,^8^ poderiam estar, de forma direta, relacionados com uma possível perda de peso, porém não houve nos 30 dias de jejum alterações significantes no peso. Também não ocorreram alterações no perfil lipídico e glicídico. Alguns estudos de JI consideram que os benefícios cardiovasculares e metabólicos se devem devido a perda de massa gorda e melhora dos lipídios com redução de triglicérides e da LDL, além da melhora do estresse oxidativo.^9^

Neste estudo houve somente diminuição significativa dos níveis de PCR, pois o período de 30 dias é não é suficiente para ocorrerem alterações significantes do ponto de vista metabólico. Patikorn et al.,^10^ em extensa metanálise de estudos com JI (total de 130 estudos) verificaram que a média de tempo era 2-5 meses, com achados expressivos na redução do peso corpóreo e melhora nas condições metabólicas, aliados à queda pressórica.^10^

Dermici et al.,^8^ foram muito modestos nas conclusões do trabalho. Avaliar o SRAA e o sistema nervoso simpático em humanos hipertensos é importante também para gerar hipóteses para a realização de trabalhos futuros. Assim, com desenhos de estudos mais acurados talvez possamos responder com fundamentos mais sólidos às questões da complexa fisiopatologia da hipertensão arterial. O tratamento da hipertensão arterial se fundamenta principalmente em bloqueios nos diversos níveis da SRAA, além de reduzir a atividade simpática.^11^

Por isso o conhecimento desta dinâmica é fundamental no manuseio terapêutico dos fármacos que agem no sistema cardiovascular, principalmente no tratamento da hipertensão e em outras situações que envolvam o SRAA como a insuficiência cardíaca.
