# Supplementary material for: O Jejum Intermitente e a Redução da Pressão Arterial: Mecanismos Relacionados
Source: Arq Bras Cardiol. 2023 Jun 2;120(5):e20230265. [Article in Portuguese] doi: 10.36660/abc.20230265 (PMC10263398; doi:10.36660/abc.20230265)
Supplement: Translation

# Translation: Intermittent Fasting and Blood Pressure Reduction: Related Mechanisms

**Authors:** Rui Póvoa

**Affiliations:** 1 Brasil Universidade Federal de São Paulo, São Paulo, SP – Brasil

**Keywords:** Cardiovascular Diseases/mortality, Obesity, Hypertension, Fasting, Caloric Restriction, Weight Loss

Obesity and an inadequate diet are modifiable risk factors that play an important role in the increase of cardiovascular diseases. They have an estimated 13% attributable risk of cardiovascular mortality.^1^ Several dietary interventions can improve this risk, most notably calorie restriction, which is linked to weight loss, improved insulin sensitivity, and reduced blood pressure.^2 , 3^

Intermittent fasting (IF) is a dietary intervention similar to calorie restriction, as it uses the principle of restricting food intake. The two most comprehensive types are alternate daytime fasting (24 hours followed by a 24-hour eating period) and time-restricted fasting (16-20 hour variations).

Some human studies have shown cardiovascular benefits and pressure reduction with IF. Sutton et al., in 2018, evaluated the reduction in blood pressure with the 18-hour IF, finding a reduction of 11 ± 4 mmHg for systolic blood pressure after six weeks and 10 ± 4 mmHg for systolic blood pressure diastolic.^5^

The mechanisms that propose to explain these pressure variations are complex and multifactorial, like the very genesis of hypertension. However, the decrease in sympathetic activity and the increase in parasympathetic tonus, in addition to the reduction in the activity of the renin-angiotensin-aldosterone system (RAAS), are the most concurrent phenomena for this pressure drop.^6^ Experimental studies in rats, where it is possible to control the variables better, point in this direction, where the IF corrects the autonomic imbalance, reducing the pressure and vagal elevation associated with lower levels of inflammatory cytokines.^7^

However, controlled studies evaluating these systems and their interactions with humans are very scarce, with results that do not allow a coherent explanation.

Dermirci et al.^8^ analyzed the influence of IF on blood pressure with an extensive assessment of RAAS activity markers and sympathetic activity.^8^ The work involved a significant number of hypertensive patients with evaluations before and after the IF, and the results were very interesting, especially in markers related to the development of hypertension. After 15-16 hours of fasting for 30 days, they found significant reductions in angiotensin I converting enzyme (ACE) and angiotensin II (Ang-II) and an equally significant increase in angiotensin I (ang-I). Allied with these results, there was a statistically significant reduction in both systolic and diastolic blood pressure, including at night. Consistent results that meet the pathophysiological aspects of arterial hypertension.

We know that losing body weight is an important factor for blood pressure reduction and that IF can provide these benefits in the long run.

Evidently, the effects of IF in the study by Dermirci et al.^8^ could be directly related to a possible weight loss, but there were no significant changes in weight during the 30 days of fasting. There were also no alterations in the lipid and glucose profiles. Some IF studies consider that the cardiovascular and metabolic benefits are due to the loss of fat mass and improvement in lipids with a reduction in triglycerides and LDL, in addition to the improvement in oxidative stress.^9^

In this study, there was only a significant decrease in CRP levels, as the 30-day period is not enough for significant changes to occur from the metabolic point of view. Patikorn et al.,^10^ in an extensive meta-analysis of studies with IF (a total of 130 studies), found that the average time was 2-5 months, with significant findings in the reduction of body weight and improvement in metabolic conditions, combined with a drop in blood pressure.^10^

Dermici et al.^8^ were very modest in their conclusions. Assessing the RAAS and the sympathetic nervous system in hypertensive humans is also important to generate hypotheses for future work. Thus, with more accurate study designs, we may be able to respond with more solid foundations to the questions of the complex pathophysiology of arterial hypertension. The treatment of arterial hypertension is mainly based on blocks at different levels of the RAAS, in addition to reducing sympathetic activity.^11^

Therefore, knowledge of this dynamic is fundamental in the therapeutic management of drugs that act on the cardiovascular system, especially in the treatment of hypertension and in other situations involving RAAS, such as heart failure.
